# Evaluation of the Stability, Bioavailability, and Hypersensitivity of the Omega-3 Derived Anti-Leukemic Prostaglandin: Δ^12^-Prostaglandin J_3_

**DOI:** 10.1371/journal.pone.0080622

**Published:** 2013-12-02

**Authors:** Avinash K. Kudva, Naveen Kaushal, Sonia Mohinta, Mary J. Kennett, Avery August, Robert F. Paulson, K. Sandeep Prabhu

**Affiliations:** 1 Department of Veterinary and Biomedical Sciences, The Pennsylvania State University, University Park, Pennsylvania, United States of America; 2 The Department of Microbiology and Immunology, Cornell University, Ithaca, New York, United States of America; University of South Alabama Mitchell Cancer Institute, United States of America

## Abstract

Previous studies have demonstrated the ability of an eicosapentaenoic acid (EPA)-derived endogenous cyclopentenone prostaglandin (CyPG) metabolite, Δ^12^-PGJ_3_, to selectively target leukemic stem cells, but not the normal hematopoietic stems cells, in *in vitro* and *in vivo* models of chronic myelogenous leukemia (CML). Here we evaluated the stability, bioavailability, and hypersensitivity of Δ^12^-PGJ_3_. The stability of Δ^12^-PGJ_3_ was evaluated under simulated conditions using artificial gastric and intestinal juice. The bioavailability of Δ^12^-PGJ_3_ in systemic circulation was demonstrated upon intraperitoneal injection into mice by LC-MS/MS. Δ^12^-PGJ_3_ being a downstream metabolite of PGD_3_ was tested *in vitro* using primary mouse bone marrow-derived mast cells (BMMCs) and *in vivo* mouse models for airway hypersensitivity. ZK118182, a synthetic PG analog with potent PGD_2_ receptor (DP)-agonist activity and a drug candidate in current clinical trials, was used for toxicological comparison. Δ^12^-PGJ_3_ was relatively more stable in simulated gastric juice than in simulated intestinal juice that followed first-order kinetics of degradation. Intraperitoneal injection into mice revealed that Δ^12^-PGJ_3_ was bioavailable and well absorbed into systemic circulation with a *C_max_* of 263 µg/L at 12 h. Treatment of BMMCs with ZK118182 for 12 h resulted in increased production of histamine, while Δ^12^-PGJ_3_ did not induce degranulation in BMMCs nor increase histamine. In addition, *in vivo* testing for hypersensitivity in mice showed that ZK118182 induces higher airways hyperresponsiveness when compared Δ^12^-PGJ_3_ and/or PBS control. Based on the stability studies, our data indicates that intraperitoneal route of administration of Δ^12^-PGJ_3_ was favorable than oral administration to achieve effective pharmacological levels in the plasma against leukemia. Δ^12^-PGJ_3_ failed to increase histamine and IL-4 in BMMCs, which is in agreement with reduced airway hyperresponsiveness in mice. In summary, our studies suggest Δ^12^-PGJ_3_ to be a promising bioactive metabolite for further evaluation as a potential drug candidate for treating CML.

## Introduction

Prostaglandins (PGs) are cyclooxygenase-derived products of long chain polyunsaturated fatty acids (PUFA), which exhibit diverse biological functions depending on their structure, location, and concentration [1]. PGE_1_, PGI_2_ and PGF_2α_ possess therapeutic potential with limited stability at various physiological and non-physiological conditions [2]–[4]. Younger *et al*, reported that saline solution of PGE_1_, a vasodilator and smooth muscle relaxant widely used for treatment of erectile dysfunction, was found to be less stable at 37°C, pH 7.4, but the stability was significantly improved at 4°C [2]. Pifer *et al* demonstrated that PGI_2_ was more stable in the presence of human plasma than in a buffer devoid of plasma protein at pH 7.55, where binding of PGI_2_ to albumin stabilized the molecule from degradation [4].

**Figure 1:**
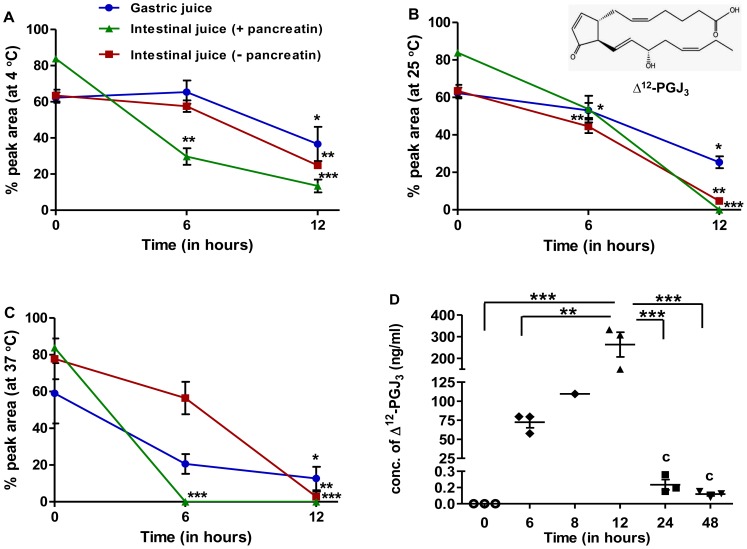
Study on stability and bioavailability of Δ^12^-PGJ_3_. (A–C) Δ^12^-PGJ_3_ (0.5 µg/mL) was incubated in artificial gastric and intestinal juice (with or without pancreatin) for various time intervals and temperatures (4°C (A), 25°C (B), 37°C (C) and estimated for % of Δ^12^-PGJ_3_ remaining by HPLC. Significant differences between concentrations Δ^12^-PGJ_3_ at different time points as compared to t = 0 are indicated by *p≤0.05; **p≤0.01; ***p≤0.001 respectively. Data shown are the mean ± SEM (n = 3). (D) Time course analysis for bioavailability of Δ^12^-PGJ_3_ in mouse plasma. Δ^12^-PGJ_3_ (0.025 mg/kg body weight) was injected intraperitoneally into C57BL/6 mice. The concentration of Δ^12^-PGJ_3_ in plasma was measured at indicated times post injection by LC-MS/MS. The chemical structure of Δ^12^-PGJ_3_ is shown in the inset of panel B. All data shown are mean ± SEM (n = 3) and statistical significance represented as *- *p*≤0.05; **- *p*≤0.01; ***- *p*≤0.001 respectively.

Cyclopentenone prostaglandins (CyPGs) are metabolites of PGs that possess an alkylidenecyclopentenone ring and are produced by non-enzymatic dehydration and isomerization of PGD_2_ and PGD_3_ from arachidonic acid (ARA) and eicosapentaenoic acid (EPA), respectively [5], [6]. Of these, ARA-derived Δ^12^-prostaglandin J_2_ (Δ^12^-PGJ_2_) and 15-deoxy-Δ^12,14^-prostaglandin J_2_ (15d-PGJ_2_) have been characterized for their antineoplastic, antiviral, and anti-inflammatory activities [5]. Recently, we described the role of a CyPG metabolite, Δ^12^-prostaglandin J_3_ (Δ^12^-PGJ_3_) (Fig. 1B inset) derived from EPA, a marine omega-3 (n-3) PUFA, to alleviate the progression of leukemia in a murine model of chronic myelogenous leukemia (CML) [7]. It was found that Δ^12^-PGJ_3_ selectively targeted leukemia stem cells (LSC) for apoptosis in the spleen and bone marrow, but spared the normal hematopoietic stem cells (HSC) [7]. Although the mechanisms of apoptosis are currently being investigated, it is known that CyPGs primarily act *via* two different mechanisms that includes binding to two G protein-coupled receptors, DP1 and CRTH2 (DP2) [8], [9], and/or *via* covalent modification of specific cysteine residues in a subset of cellular proteins [10]. Synthetic agonists of the DP receptor such as ZK118182 have been used to treat high intraocular pressure in glaucoma [11]. On the other hand, activation of DP receptor(s) have been associated with increasing risk of airway hypersensitive responses mediated through CRTH2 in the degranulation of mast cells and eosinophils [11], [12]. Therefore, considering the evolving pharmacological role of Δ^12^-PGJ_3_, we deemed it essential to further assess the stability under physiological conditions and possible toxicities in terms of hypersensitivity that essentially dictates its efficacy as a potential therapeutic for CML.

Here we have examined the thermal stability and pH stability of Δ^12^-PGJ_3_ under simulated conditions followed by its bioavailability *in vivo* and the likelihood of any potential risk of hypersensitivity of Δ^12^-PGJ_3_ to cause degranulation of mast cells. Our studies indicate that Δ^12^-PGJ_3_ was stable and bioavailable upon intraperitoneal administration in mice. Furthermore, *in vitro* and *in viv*o experiments clearly show that Δ^12^-PGJ_3_ differed from the synthetic agonist of DP1 receptor, ZK118182, in terms of its ability to induce airways hyperresponsiveness, release of histamine, and IL-4 expression, which are all associated with hypersensitivity. In summary, these studies should pave way for in-depth immunotoxicological studies of Δ^12^-PGJ_3_ for use as a therapeutic agent in CML.

## Materials and Methods

### Materials

Artificial gastric juice (hydrochloric acid 1%, pepsin (1∶3000) <1%, water 98%), artificial intestinal juice (potassium phosphate, monobasic <1%, sodium hydroxide 50%, <1%, water 99%) and pancreatin (a crude mixture of digestive enzymes composed of amylases, lipases, and proteases) were purchased from ScholAR® chemistry (Rochester, NY). ZK118182 was purchased from Cayman Chemicals (Ann Arbor, MI) and used without any further purification. Δ^12^ PGJ_3_ was prepared as described earlier from our laboratory [7]. The purity of Δ^12^-PGJ_3_ was confirmed by LC-MS/MS to be ≥99%. Toluidine blue, ionomycin, and all HPLC grade-solvents used were from Sigma-Aldrich (St. Louis, MO). Histamine EIA kit was purchased from Cayman Chemical (Ann Arbor, MI).

### Methods

#### Measurement of the stability of Δ^12^-PGJ_3_ in simulated gastric and intestinal juices

Aliquots of Δ^12^-PGJ_3_ (0.5 µg) in ethyl acetate were evaporated to dryness under a stream of N_2_. The dry residue was dissolved in 1 mL of simulated gastric or intestinal juice (with or without pancreatin) and incubated at 4°C, 25°C and 37°C for different time durations (0, 6 and 12 h). Following incubation, the solutions were extracted with two volumes of hexane-diethyl ether (1∶1) mixture twice after acidification with HCl (6 N) to ∼pH 3.0. The extracts were pooled and evaporated under a stream of N_2_, re-constituted with mobile phase (acetonitrile: water = 30∶70 v/v, 0.1% v/v acetic acid, 0.01% triethylamine), and analyzed by HPLC.

#### Analysis by liquid chromatography

Samples were chromatographically analyzed using Dynamax C_18_ column (10×250 mm; pore size = 300 Å) on a Beckman system Gold® HPLC. The detector was set at 244 nm. A gradient solvent system was applied at a flow rate of 1 mL/min using mobile phase consisting of solvent-A (acetonitrile/water (30∶70 v/v), 0.1% acetic acid, and 0.01% TEA) and solvent-B (acetonitrile (100%), 0.1% acetic acid, and 0.01% TEA). At injection and 10 min thereafter, the mobile phase was at 100% solvent-A. A linear gradient was achieved over a period of 40 min to 60% solvent-B and further continued at that composition for another 10 min. The peak area was calculated and stability of PG’s at each designated time interval was expressed as the percentage of its original concentration (% remaining). All studies were performed in triplicate.

#### Determination of the half-life of Δ^12^-PGJ_3_ in simulated gastric and intestinal juices

The rate constant and half-life for Δ^12^-PGJ_3_ was determined using Arrhenius equation (first order kinetics) as given below:
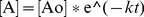
(1)


Where, [Ao] and [A] are the initial and final concentration of the compound, ê(−kt) is the exponential product of rate constant (*k*) and time (t).
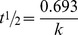
(2)


Where, ‘t_1/2_’ is the amount of time needed for a reactant concentration to decrease by half compared to its initial concentration at any given condition and ‘*k*’ is the reaction rate constant.

### Ethics Statement

All animal work reported here has been conducted according to United States Animal Welfare Act (Public Law 99–198). These studies were pre-approved by IACUC (#40679 and #2013–0014) at The Pennsylvania State University and Cornell University, respectively. Accordingly the authors have taken all steps to ameliorate suffering.

### Bioavailability of Δ^12^-PGJ_3_ in Mice

Δ^12^-PGJ_3_ (0.025 mg/kg body weight) was intraperitoneally injected into C57BL/6 mice (6–8 month old, Charles River Laboratories International, Inc., USA). Post 6, 12, 24 and 48 h of injection, blood (500 µL) was collected in an EDTA coated collection vial (CAPIJECT®, Terumo Medical Corp, USA) by retro-orbital venous puncture. A group comprising of PBS injected mice were designated as vehicle control. The plasma was separated by centrifugation at 1200 x*g* for 15 min and Δ^12^-PGJ_3_ was extracted by using a Sep-Pak® C18 classic cartridge (Waters corporation, USA). Briefly, plasma (100 µL) was diluted to 2 mL using PBS and acidified with 6 N HCl to pH 3. The C_18_ cartridge was cleaned with methanol (5 mL) followed by 5 mL of PBS and then plasma was passed through twice. The column was washed with PBS and hexane (5 mL each) and the bound PGs were eluted with methanol (5 mL). This fraction was evaporated to dryness under a stream of N_2_ and re-dissolved with 100 µL solvent-A and analyzed by LC-MS/MS as described below.

### LC-MS/MS-MRM Analysis

The HPLC system consisted of LC-20AD UFLC pumps with a SIL-20AC autosampler (Shimadzu Corporation, Columbia, MD). A Luna (Phenomenex, Torrance, CA) phenyl-hexyl analytical column (2×150 mm, 3 µm) developed with a 30 min isocratic elution with methanol/water (70∶30 v/v) containing 0.1% acetic acid at a flow rate of 150 µL/min was used for the quantitation of Δ^12^-PGJ_3_. The injection volume was 50 µL. Negative ion electrospray tandem mass spectrometric analysis was carried out using API 2000 triple quadruple mass spectrometer (AB Sciex, Foster city, CA) at unit resolution with multiple reaction-monitoring mode (MRM). The source temperature was at 450°C, electrospray voltage was −4500 V and the declustering potential was set at −16 V. Nitrogen was used as collision gas at −20 eV and the dwell time was 150 ms/ion. During MRM, Δ^12^-PGJ_3_ was measured by recording the signal for the transition of the deprotonated molecule of *m/z* 331 to the most abundant fragment ion with *m/z* 269. Data were acquired and analyzed using Analyst software program version 1.5 (AB Sciex, Foster city, CA).

### *In vitro* Testing for Drug Hypersensitivity using Murine Bone Marrow-derived Mast Cells (BMMCs)

#### a) Culturing murine BMMCs

BMMCs were cultured as described previously with some modifications [13]. Briefly, femoral marrow was extracted from C57BL/6 mice (∼4 month old) and cultured in DMEM supplemented with 10% FBS (HyClone, Logan, UT), 100 U/mL penicillin, 100 µg/mL streptomycin, 100 µM non-essential amino acids, 1 mM sodium pyruvate, 2 mM glutamine (Invitrogen, Grand Island, NY), 50 µM 2-mercaptoethanol (Sigma-Aldrich, St. Louis, MO) and recombinant murine IL-3 (rmIL-3; 20 ng/mL; Peprotech, Rocky Hill, NJ). Cells were passaged every three days in fresh medium and used for experiments after 5–6 weeks. The mature BMMCs were stained with toluidine blue and assessed histochemically for the maturity of mast cells and found to be greater that 95%. These cells were utilized for the experiments described below.

#### b) Cytological analysis

BMMCs (1×10^6^ cells/mL) cultured in growth media were treated for 6 h and 12 h with PBS, Δ^12^-PGJ_3_ (0.1 µM), or ZK118182 (0.1 µM). As a positive control, ionomycin (1 µM) was added to the cells for 1 h. Following incubation, the cells were fixed on glass slides using methanol for 30 min followed by staining with toluidine blue for 5 min, rinsed with Milli-Q water, and examined microscopically for any signs of degranulation.

#### c) Histamine release assay

The supernatant and cell lysates from BMMCs subjected to various treatments (as described above) were used in the quantitation of histamine using the histamine EIA kit. The amount of histamine released was expressed as the ratio of percent of histamine released in the supernatant compared to the intracellular histamine content.

#### d) Analysis for inflammatory markers by real-time PCR

Total RNA from treated BMMCs (as described above) was isolated using Trizol reagent according to manufacturer’s instructions (Life Technologies, Grand Island, NY). Reverse transcription-PCR was performed using 1 µg of total RNA with random primers using the High Capacity cDNA Reverse Transcription Kit (ABI- Life technologies, Grand Island, NY) in a PTC-100 thermal cycler (MJ Research Inc, Waltham, MA) as per the manufacturer’s protocol. Real-time PCR was performed using Taqman probes for interferon-γ (IFN-γ), TNF-α, IL-13, and IL-4 (ABI-Life Technologies, Grand Island, NY). Real-time PCR was performed according to manufacturer’s instructions using PerfeCTa® qPCR kit (Quanta biosciences, Gaithersburg, MD) on ABI 7300 real-time PCR (ABI- Life Technologies, Grand Island, NY). Results were expressed as 2^−▵▵CT^ that is the expression of target gene relative to the house-keeping gene (GAPDH) and normalized to the negative control (PBS treated cells). All studies were performed in triplicate.

### *In vitro* Testing for Drug Hypersensitivity in Mice

#### a) Acute and chronic drug toxicity test

Mice (C57BL/6, 6–8 months old) were injected intraperitoneally with Δ^12^-PGJ_3_ (0.025 mg/kg body weight) or sterile PBS (300 µL) and euthanized after 6 h or 12 h of injection. The lung tissues were extracted, fixed, sectioned, and stained with hematoxylin and eosin (H&E) for histological examination. Furthermore, inflammatory gene expression in alveolar tissue was also examined using RT-PCR as described earlier and histamine levels in the serum measured using histamine EIA kit as described earlier.

In a separate experiment, mice were treated with Δ^12^-PGJ_3_ and ZK118182 at two doses (0.025 mg/kg body weight/day and 0.050 mg/kg body weight/day) intraperitoneally for two weeks, following which drug hypersensitivity response was determined by evaluating the histamine levels in the plasma. In addition, a complete blood count (CBC) and tests for blood urea nitrogen (BUN), alanine aminotransferase (ALT), and aspartate aminotransferase (AST) were carried out at the Centralized Biological Laboratory, The Pennsylvania State University, University Park, PA. Cytokine analysis was carried-out with the plasma using a mouse Th1/Th2 9-plex ultra-sensitive multi-spot cytokine panel (Meso Scale Discovery, Gaithersburg, MD). Lung tissues from the treated mice were extracted, fixed, sectioned, and stained with H&E for histological examination.

#### b) Measurement of Airways Hyperresponsiveness (AHR)

Mice (C57BL/6, 6–8 weeks old) were injected intraperitoneally with Δ^12^-PGJ_3_; ZK118182 at two doses (0.025 mg/kg body weight/day and 0.050 mg/kg body weight/day) or sterile PBS (300 µL) for seven days. After twenty-four hours of final challenge, AHR was analyzed using FlexiVent apparatus (SCIREQ USA Inc., Tempe, AZ) as described [14], [15]. Briefly, airway hyperresponsiveness was measured using a mechanical ventilator apparatus in response to PBS or increasing doses of aerosolized sympathomimetic methacholine from 1 mg/mL to 100 mg/mL. The results were plotted as function of respiratory system resistance (Rrs) values (cm H_2_O/ml/sec) versus methacholine concentration. Subsequent to AHR measurements, real-time PCR was carried-out on the alveolar tissue to analyze the expression of inflammatory genes.

### Statistics

All results are expressed as mean ± SEM. An un-paired two-tailed t-test was used to compare the mean for each treatment group with the mean of the control group and one-way ANOVA (Tukey multiple comparison method) or two-way ANOVA was performed in order to compare various treatment groups within *in vivo* studies using GraphPad Prism 5.0 program (GraphPad software Inc., San Diego, CA). *p* values ≤0.05 were considered as statistically significant (*p≤0.05; **p≤0.01; ***p≤0.001).

## Results

### Effect of Temperature and pH on the Stability of Δ^12^-PGJ_3_ under Simulated Conditions

Analysis of extraction efficiency of Δ^12^-PGJ_3_ from gastric and intestinal juice (with or without pancreatin) indicated the recovery in the range of 60–80% at t = 0. A time-dependent reduction in Δ^12^-PGJ_3_ levels was seen in both simulated gastric and intestinal juice (Fig. 1 A–C) upon incubation at 4°C, 25°C, and 37°C.

After 12 h of incubation at 4°C, Δ^12^-PGJ_3_ levels were reduced to ∼50% in the presence of gastric and intestinal juice (−pancreatin). However, addition of pancreatin led to a further decrease in the extractable Δ^12^-PGJ_3_ with only a <25% final recovery. At 25°C, ∼50% of Δ^12^-PGJ_3_ was recovered after 12 h incubation in gastric juice, but only <5% was recovered upon incubation with intestinal juice (+/− pancreatin). Incubation at 37°C resulted in ∼30% recovery of Δ^12^-PGJ_3_ after 12 h incubation in gastric juice and <5% recovery in intestinal juice (−pancreatin). However, after 6 h of incubation, >50% loss was observed in gastric juice, which further reduced to ∼10% at the end of 12 h. In the case of Δ^12^-PGJ_3_ incubated in intestinal juice (without pancreatin) at t = 6 h, there was <25% loss in concentration and a significant reduction was observed at the end of 12 h with only a <5% recovery. Addition of pancreatin to the intestinal juice had a significant impact on the stability of Δ^12^-PGJ_3_ with no extractable free Δ^12^-PGJ_3_ after 6–12 h of incubation at 37°C. Thus, our results suggest that the oral route of administration may be least effective for Δ^12^-PGJ_3_.

Based on stability studies of Δ^12^-PGJ_3,_ it was found that under given conditions the rate constant and half-life did not change with time and hence they were considered to follow first-order kinetics of degradation (data not shown). The half life of Δ^12^-PGJ_3_ was compared in simulated gastric and intestinal juice (in the presence or absence of pancreatin) at three separate temperatures, 4°C, 25°C, and 37°C. The kinetics of degradation of Δ^12^-PGJ_3_ was faster at higher temperatures and under increasing pH (gastric to intestinal). At 37°C, the t_0.5_ in gastric juice, intestinal juice (−pancreatin), and intestinal juice (+pancreatin) were 5.5, 3.3, and 1 h, respectively (Table 1). On the other hand, Δ^12^-PGJ_3_ was relatively more stable at 4°C. Furthermore, a change in pH towards basic conditions increased the degradation of Δ^12^-PGJ_3_ indicating poor stability in the presence of pancreatic enzymes (Table 1).

**Table 1:** Calculated half-life (t_1/2_) and rate constant (k) for Δ^12^-PGJ_3_ #.

	Incubated temperatures		
Samples	4°C	25°C	37°C
**Gastric juice**	13.1 h [1.47E-05]	7 h [2.76E-05]	5.5 h [3.52E-05]
**Intestinal (−Pancreatin)**	9.7 h [1.97E-05]	3.5 h [5.45E-05]	3.3 h [5.75E-05]
**Intestinal (+Pancreatin)**	5.9 h [3.26E-05]	1.2 h [8.56E-05]	1 h [8.9E-05]

# Half-life (t_1/2_) and Reaction rate constant (k) (indicated in square brackets) were calculated based on Arrhenius equation-first order kinetics for Δ^12^-PGJ_3_ at various incubated temperatures and simulated physiological conditions.

### Bioavailability of Δ^12^-PGJ_3_ upon Intra-peritoneal Administration

Bioavailability is described as the amount of “unmodified free drug” that is present in the systemic circulation when introduced through non-systemic routes, including intraperitoneal route of administration. In the present study, we used a dose of 0.025 mg/kg/day of Δ^12^-PGJ_3_ that was previously reported to be effective in targeting LSCs in two *in vivo* models of leukemia [7]. Here, we examined the systemic levels of Δ^12^-PGJ_3_ for up to 48 h following a single intra-peritoneal administration. Assuming that the Δ^12^-PGJ_3_ was uniformly absorbed by mice (that were strain, sex, and age-matched) and that average total volume of blood in each mouse was about ∼8% of its body weight [16], we determined the extractable amount of Δ^12^-PGJ_3_ in the plasma in mice as a function of time. LC-MS/MS-MRM analysis indicated that upon 12 h of injection of 0.025 mg/kg, only ∼45% of the total Δ^12^-PGJ_3_ was found in systemic circulation with a C_max_ of 263 µg/L that decreased to baseline levels by 48 h (Fig. 1D). These results indicate that Δ^12^-PGJ_3_ was stable and well absorbed into systemic circulation when injected intra-peritoneally. The peak plasma concentrations of Δ^12^-PGJ_3_ were significantly higher than the IC_50_ of 7–10 nM for LSC apoptosis [7].

### Treatment of BMMCs with Δ^12^-PGJ_3_ does not Increase Histamine Production

#### a) Cytological examination of BMMCs upon treatment with Δ^12^-PGJ_3_

**Figure 2:**
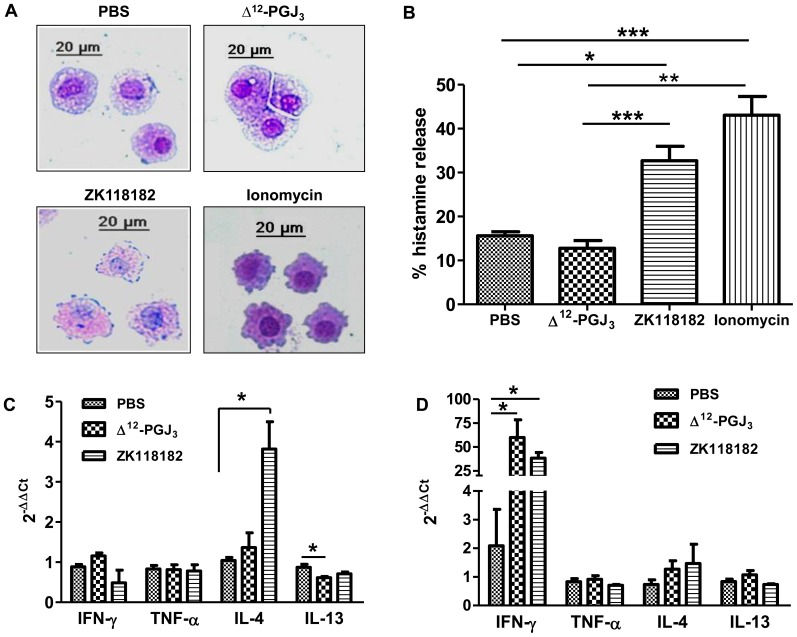
*In vitro* effect of Δ^12^-PGJ_3_ and ZK118182 on BMMCs. (A) Cytological evaluation for drug hypersensitivity upon treatment of BMMCs with Δ^12^-PGJ_3_ and ZK118182. Cultured BMMCs (1×10^6^ cells/mL) were treated with Δ^12^-PGJ_3_ and ZK118182 at 0.1 µM for 12 h, followed by fixation and staining with toluidine blue. BMMCs treated with ionomycin (1 µM) for 1 h was used as a positive control. All treatments were carried out in triplicate and a representative image is shown (magnification: 32X). (B) The cells and media treated as mentioned above were used for the estimation of histamine (total and released) using EIA method. (C–D) Real time PCR analysis was carried-out for pro-inflammatory gene markers upon treatment of BMMCs with PBS, ΔΔ^12^-PGJ_3_ and ZK118182 as mentioned above (C-6 h; D-12 h). The data shown are mean ± SEM (n = 3) and statistical significance are represented as *- *p*≤0.05; **- *p*≤0.01; ***- *p*≤0.001 respectively.

Primary BMMCs treated with PBS or Δ^12^-PGJ_3_ (0.1 µM) for 6 h and 12 h showed uniform presence of metachromatic granules when stained with toluidine blue with no morphological changes. However, BMMCs treated with ZK118182 showed prominent loss in cellular integrity with degranulation upon 6 h and 12 h of treatment (Fig. 2A). As a control, ionomycin treatment for 1 h led to extensive degranulation and loss in cellular integrity.

#### b) Histamine release assay

Histamine release assay was carried out on the total BMMC lysates as well as in the corresponding culture media supernatants in the above treatments (Fig. 2B). The results were calculated as a percent of histamine released into the culture media, taking into account the intracellular histamine content. Treatment of BMMCs with Δ^12^-PGJ_3_ and ZK118182 for 6 h failed to show any significant increase in histamine release when compared to PBS-treated group. However, incubation of BMMCs with ZK118182 for 12 h induced a ∼3.5 fold increase in histamine release into the supernatant, while Δ^12^-PGJ_3_ and PBS treatments failed to induce any response. Ionomycin treatment led to ∼4.5 fold increase in histamine release compared to PBS-treated cells.

#### c) Real-time PCR analysis

Real-time PCR was carried out to analyze expression of IFN-γ, TNF-α, IL-4 and IL-13 upon treatment with PBS, Δ^12^-PGJ_3_ (0.1 µM), or ZK118182 (0.1 µM). Treatment with ZK118182 for 6 h increased IL-4 by ∼4 fold (Fig. 2C). However, no significant changes were seen in the expression of IFN-γ, TNF-α, and IL-13 by Δ^12^-PGJ_3_ or ZK118182 when compared to PBS control. Interestingly, upon 12 h of treatment, a significant increase in IFN-γ expression was noted in Δ^12^-PGJ_3_ (∼60 fold) and ZK118182 (∼40 fold) treated cells (Fig. 2D).

### Toxicological Evaluation of Δ^12^-PGJ_3_ *in vivo*

#### a) Acute and chronic drug toxicity tests in mice

**Figure 3:**
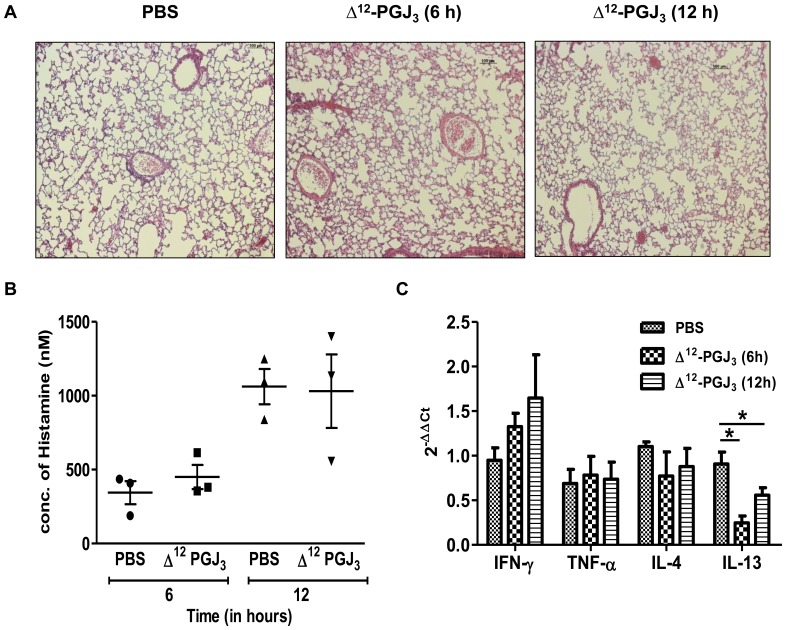
*In vivo* evaluation for hypersensitivity by acute toxicity test. (A) Δ^12^-PGJ_3_ (0.025 mg/kg body weight) was injected intraperitoneally into C57BL/6 mice. Post 6 h and 12 h the mice were sacrificed and the lungs extracted, fixed and stained by H&E. Mice treated with PBS for 12 h was used as a placebo control. All treatments were carried out in triplicates and a representative image has been shown (magnification: 10X). (B) Histamine release assay was carried out on the plasma from the mice treated as mentioned above by EIA method. (C) A real-time PCR analysis was carried out for pro-inflammatory gene markers upon treatment of mice with PBS and Δ^12^-PGJ_3_ as mentioned above. The data shown are mean ± SEM (n = 3) and statistical significance are represented as *- *p*≤0.05.

Histological examination was conducted on the alveolar tissue of mice treated with Δ^12^- PGJ_3_ for 6 h and 12 h (Fig. 3A). Blinded H&E sections were scored based on the infiltration of PMNs and macrophages, presence of peribronchiolar and perivascular lymphoid aggregates, airway hyperplasia, and necrosis. Based on these criteria, examination of tissues indicated no significant changes in the gross pathology upon treatment with Δ^12^-PGJ_3_ (data not shown). In addition, no significant changes in the histamine content in the plasma of Δ^12^-PGJ_3_ treated animals were seen when compared to the PBS-treated control mice (Fig. 3B). Furthermore, real-time PCR-based expression analysis of the alveolar tissue indicated a time-dependent increase in IFN-γ, while IL-4 and IL-13 were decreased and TNF- α was not affected upon treatment with Δ^12^-PGJ_3_ (Fig. 3C).

**Figure 4:**
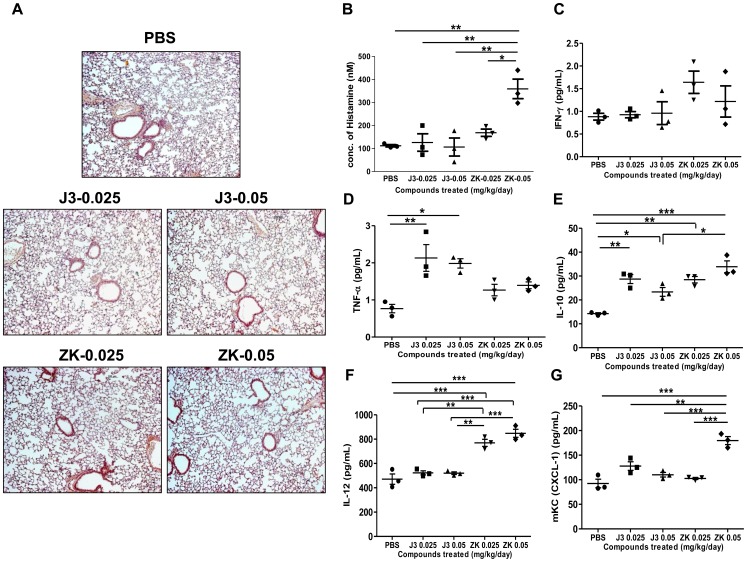
*In vivo* evaluation for hypersensitivity by chronic toxicity test. (A) Δ^12^-PGJ_3_ (0.025 and 0.05 mg/kg body weight) and ZK118182 (0.025 and 0.05 mg/kg body weight) were injected intraperitoneally into C57BL/6 mice. Post two weeks of treatment, the alveolar tissue was extracted, fixed, and stained with H&E. Mice treated with PBS were used as placebo control. All treatments were carried out in triplicates and a representative image has been shown (magnification: 10X). (B) Histamine release assay was carried out on the plasma from the mice treated as mentioned above by EIA method. (C–G) Multi-array Th1/Th2 cytokine analysis was carried-out using the plasma, upon treatment of mice as mentioned above. The figures shown are for: (C) IFN-γ, (D) TNF-α, (E) IL-10, (F) IL-12 and (G) mKC (CXCL-1). The data shown are mean ± SEM (n = 3) and statistical significance are represented as *- *p*≤0.05; **- *p*≤0.01; ***- *p*≤0.001 respectively.

In the second set of mice that were treated with multiple doses (long-term treatment for 14 days) of Δ^12^-PGJ_3_, CBC analysis revealed no significant changes in the levels of WBCs and RBCs as compared to initial levels, where ZK118182 was used for comparison (Fig. S1a–d). However, a significant increase in platelet count was observed in Δ^12^-PGJ_3_ treated mice (Fig. S1e–f). A single slide chemical analysis of the serum derived from the mice was performed as an indicator to gauge the performance of vital organs. The results revealed no significant deviation in BUN levels between the control and test groups and were found to be within the average range of 18–22 mg/dL (Fig. S1g). In addition, liver function tests for AST activity showed no significant differences between the test groups and the vehicle control; however Δ^12^-PGJ_3_ and ZK118182 treatment led to decreased levels of ALT activity in the serum suggesting lack of any overt hepatotoxicity (Fig. S1 h–i). Furthermore, tests were performed to evaluate the histamine levels in the plasma (Fig. 4B), which indicated that the control group and ^12^-PGJ_3_ (0.025 and 0.05 mg/kg/day) treated mice produced ∼100 nM of histamine post 24 h of final injection. However, mice treated with ZK118182 (0.025 mg/kg/day) produced ∼175 nM of histamine. Furthermore, mice treated with 0.05 mg/kg/day of ZK118182 nearly doubled the level of histamine (∼375 nM). Plasma cytokine analysis using a 9-plex multi-spot array kit showed a significant increase in IL-10, IL-12, and mKC (CXCL-1) upon treatment of mice with ZK118182 (at 0.025 mg/kg/day and 0.05 mg/kg/day) (Fig. 4E–G); however, treatment with Δ^12^-PGJ_3_ (at doses 0.025 mg/kg/day and 0.05 mg/kg/day) slightly elevated the levels of TNF-α, but a significant increase in IL-10 when compared to PBS treated mice was noticed (Fig. 4D–E). There was no change in the levels of IFN-γ as compared to the PBS treated mice (Fig. 4C). The histological examination of the alveolar tissues of treated mice showed no significant changes in the score (data not shown) when compared to the PBS control (Fig. 4A).

#### b) Analysis of Airways Hyperresponsiveness (AHR) in mice

**Figure 5:**
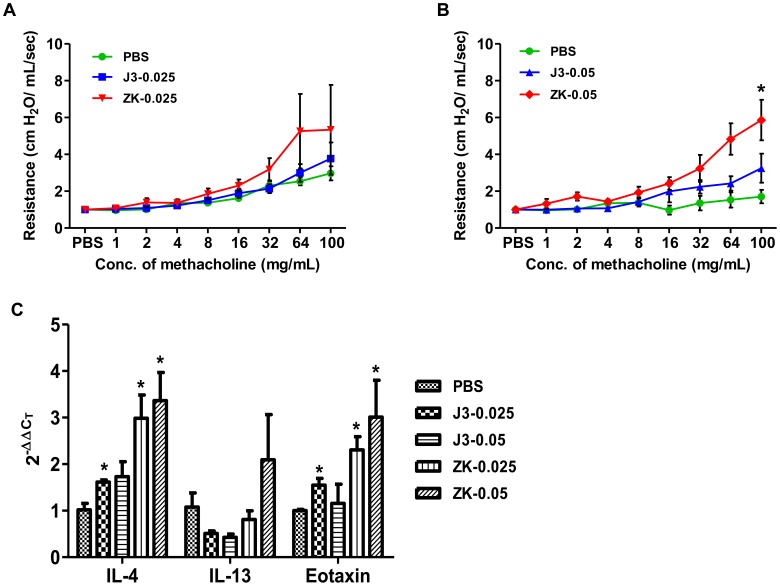
Airways hyperresponsiveness test. Δ^12^-PGJ_3_ and ZK118182 was injected intraperitoneally into C57BL/6 mice at (A) 0.025 mg/kg body weight/day and (B) 0.050 mg/kg body weight/day. Post seven days of administration, the AHR response was determined upon methacholine challenge. (C) Real-time PCR analysis was carried-out for expression of Th2 cytokines in the alveolar tissue of mice that were subjected to AHR analysis. The data shown here are mean ± SEM (n = 3) and statistical significance were calculated in comparison to (PBS) control and represented as *(*p*≤0.05).

An AHR experiment was conducted to examine any physiological changes in the respiratory function upon treatment of mice with Δ^12^-PGJ_3_ and ZK118182 (both at 0.025 mg/kg/day and 0.05 mg/kg/day). After seven days of treatment with the two compounds and placebo control (PBS), mice were examined for airway sensitivity by methacholine challenge. Mice treated with ZK118182 at 0.025 mg/kg/day and 0.05 mg/kg/day showed higher AHR when compared to PBS and Δ^12^-PGJ_3_ treated mice (Fig. 5A–B). Surprisingly, the AHR response was nearly similar in the groups treated with 0.025 mg/kg/day and 0.05 mg/kg/day of ZK118182. Real-time PCR analysis of the alveolar tissue from the above experiment for the expression of Th2 genes indicated that upon seven days of treatment with ZK118182 (0.025 mg/kg/day and 0.05 mg/kg/day), a ∼3–4 fold increase in levels of IL-4 and eotaxin was seen; while a two-fold increase in IL-13 (with 0.05 mg/kg/day) was found as compared to the PBS control (Fig. 5C). However, administration for Δ^12^-PGJ_3_ for seven days led to significant reduction in the levels of IL-13 by 2-fold (with 0.025 mg/kg/day and 0.05 mg/kg/day) with a ∼1.5 fold increase in IL-4 (with 0.025 mg/kg/day and 0.05 mg/kg/day) and eotaxin (with 0.025 mg/kg/day) as compared to PBS control.

## Discussion

Recent studies from our laboratory have demonstrated the ability of EPA-derived Δ^12^-PGJ_3_, to specifically eradicate LSCs in two models of leukemia [7]. To further expand on these results and explore the therapeutic potential of Δ^12^-PGJ_3_ as an anti-leukemic drug, we evaluated the stability, bioavailability, and hypersensitivity of this novel EPA-derived CyPG. The stability of Δ^12^-PGJ_3_ was evaluated under simulated conditions using artificial gastric juice and also in intestinal juice in the presence or absence of pancreatin. Our study based on HPLC profiling and half-life calculations indicated that the drug was stable under acidic conditions (gastric juice) and lower temperatures (4°C) for at least 6 h. However, prolonged incubation in intestinal juice (basic conditions) significantly decreased the stability of Δ^12^-PGJ_3_. Addition of pancreatin mixture in the intestinal juice further enhanced the reduction in the stability of Δ^12^-PGJ_3_. Our studies indicated that Δ^12^-PGJ_3_ followed first-order kinetics of degradation, where the rate of reaction was independent of initial concentration of the drug with an exponential reduction in concentration with time. Thus, from a therapeutic dosing point of view, oral administration of Δ^12^-PGJ_3_ appears to be the least favorable route of administration suggesting chemical modifications and/or formulation in a suitable system may likely mitigate this issue.

To evaluate bioavailability of Δ^12^-PGJ_3_, intra-peritoneal mode of administration was preferred given its efficacious ablation of LSCs in the *in vivo* models at a dose of 0.025 mg/kg/day [7]. The rate of absorption of drug upon intraperitoneal injection was found to be slow but steady with a plasma peak at 12 h post administration that accounted for ∼45% of the injected drug in systemic circulation. This result not only shows that Δ^12^-PGJ_3_ is well absorbed into systemic circulation, but also indicates its stability *in vivo*. A serum C_max_ of 263 ng/mL was achieved at 12 h that was high enough to cause apoptosis of LSCs despite the likelihood of being metabolized by reduction or conjugation as reported for 15-deoxy-Δ^12,14^-PGJ_2_ [7], [17].

ARA-derived PGD_2_ has been shown to play an important role in the pathogenesis of asthma and allergy *via* the activation of DP1 and CRTH2 (DP2) receptors [18], [19], and the fact that Δ^12^-PGJ_3_ shares structural similarity with Δ^12^-PGJ_2_, a product of PGD_2_ that is also known to bind to DP receptors [8], we evaluated the drug-induced hypersensitivity of Δ^12^-PGJ_3_ in various *in vitro* and *in vivo* models. Although we do not have any data to demonstrate Δ^12^-PGJ_3_ to act through the DP receptor *per se*, we predict that Δ^12^-PGJ_3_ could also activate the DP receptors. Therefore, we compared Δ^12^-PGJ_3_ with a potent DP-agonist ZK118182 for their ability to cause degranulation of primary BMMCs. Unlike ZK118182, Δ^12^-PGJ_3_ failed to induce any adverse reaction that corroborated with the lack of membrane blebbing or metachromatic changes as in the PBS control. Furthermore, treatment of BMMCs for 12 h with ZK118152, but not Δ^12^-PGJ_3_, significantly increased histamine production, which is consistent with the cytological observations. The reason for such a differential effect is not clear and is currently being investigated in our laboratory.

The lack of cellular response to Δ^12^-PGJ_3_ in BMMCs was further corroborated by *in vivo* studies that examined drug-induced hypersensitivity with a single dose or a two-week treatment (acute or chronic drug toxicity test). Along the same lines, histological, hematological, kidney- and liver-function tests suggested that Δ^12^-PGJ_3_ behaved differently from ZK118182. There were no significant changes in the levels of RBC and WBC upon long-term treatment (14 days) with Δ^12^-PGJ_3_ or ZK118182 when compared to untreated control. However, treatment with Δ^12^-PGJ_3_ led to a significant increase in levels of platelets without any apparent cardiotoxicity. Though the mechanism is unknown, a previous report indicated that the arachidonic acid derived 15d-PGJ_2_ increased platelets during megakaryopoesis at concentrations two-orders of magnitude higher than that is used here, and it is very likely that increasing platelet counts may be beneficial in treating thrombocytopenic patients [20]. The serum BUN and AST levels in the Δ^12^-PGJ_3_ or ZK118182 were found to be similar to that of vehicle control. However, decreased ALT activity was seen in Δ^12^-PGJ_3_ and ZK118182 groups compared to the PBS control. Since increased serum ALT activity is marker for liver injury, lack or decrease in ALT activity may be interpreted as lack of any overt hepatotoxicity. Multiplex cytokine analysis of the plasma of mice treated with ZK118182 for two weeks showed significant increase in production of IL-10, IL-12 (total) and mKC (CXCL-1). This may indicate a regulatory mechanism that could be controlling the pro- and anti-inflammatory cytokine production resulting in a balance. In such a scenario, any imbalance of such a “homeostasis” could result in side effects that have been reported earlier [11], [21]. Δ^12^-PGJ_3_, however, did not show any significant increase in any of the Th1/Th2 cytokine levels except for a 2-fold increase in TNF-α without any apparent histological lesions indicative of inflammation. Real-time PCR analysis (after 6 and 12 h treatment) further indicated decreased expression of IL-4 and IL-13 in the alveolar tissue of mice treated with Δ^12^-PGJ_3_. Although the role of IFN-γ in asthma and allergies is controversial [22], the fact that Δ^12^-PGJ_3_ treatment had no effect on IFN-γ (cytokine) production in mice, further suggests that this EPA metabolite may lack adverse responses. Along these lines, a recent report has shown that intake of fish oil can attenuate the classical allergen-induced airway inflammation and hyperreactivity in mice, which further supports our observation [23].

*In vitro* assays with BMMCs treated with ZK118182 showed increased degranulation leading to high histamine production, which corroborated well with the increased airway hyperresponsiveness of ZK118182 and increased expression of IL-4 (at 0.05 mg/kg/day), eotaxin, and IL-13 (both at 0.025 mg/kg/day and 0.05 mg/kg/day). Surprisingly, ZK118182 treatment failed to show any histological lesions in the alveolar tissue sections suggesting functional dysregulation despite active resolution.

In summary, our studies indicate the intraperitoneal route of administration of Δ^12^-PGJ_3_ is preferred over oral administration given the poor stability under basic conditions to reach a high enough C_max_ in the serum that has been shown to be effective in targeting LSCs *in vivo*. Δ^12^-PGJ_3_ did not induce histamine and IL-4 in primary murine BMMCs, which is in agreement with reduced airways hyperresponsiveness in mice. Studies are currently underway to improve the oral bioavailability and stability of Δ^12^-PGJ_3_ for future clinical trials in CML.

## Supporting Information

Figure S1 **Complete blood count analysis and organ-function tests**. PBS (A), Δ^12^-PGJ_3_ at (0.025 (B) and 0.050 (C) mg/kg body weight/day) and ZK118182 (0.025 (D) and 0.050 (E) mg/kg body weight/day) were injected intraperitoneally into C57BL/6 mice. Post 0 and 14 days of administration, the blood was collected and analyzed. Panels a, c and e indicate levels of WBC, RBC and platelets at day = 0, respectively. Panels b, d and f indicate levels of WBC, RBC and platelets at day 14, respectively. Panels g-i indicate single slide chemical analyses were carried-out after 14 days of treatments. The blood was collected and plasma analyzed for BUN (g), ALT (h) and AST (i). The data shown here are mean ± SEM (n = 3) and statistical significance represented as *(*p*≤0.05).
(TIF)
