# Supplementary figures and images for: Evaluation of the Stability, Bioavailability, and Hypersensitivity of the Omega-3 Derived Anti-Leukemic Prostaglandin: Δ12-Prostaglandin J3
Source: PLoS One. 2013 Dec 2;8(12):e80622. doi: 10.1371/journal.pone.0080622 (PMC3846793; doi:10.1371/journal.pone.0080622)

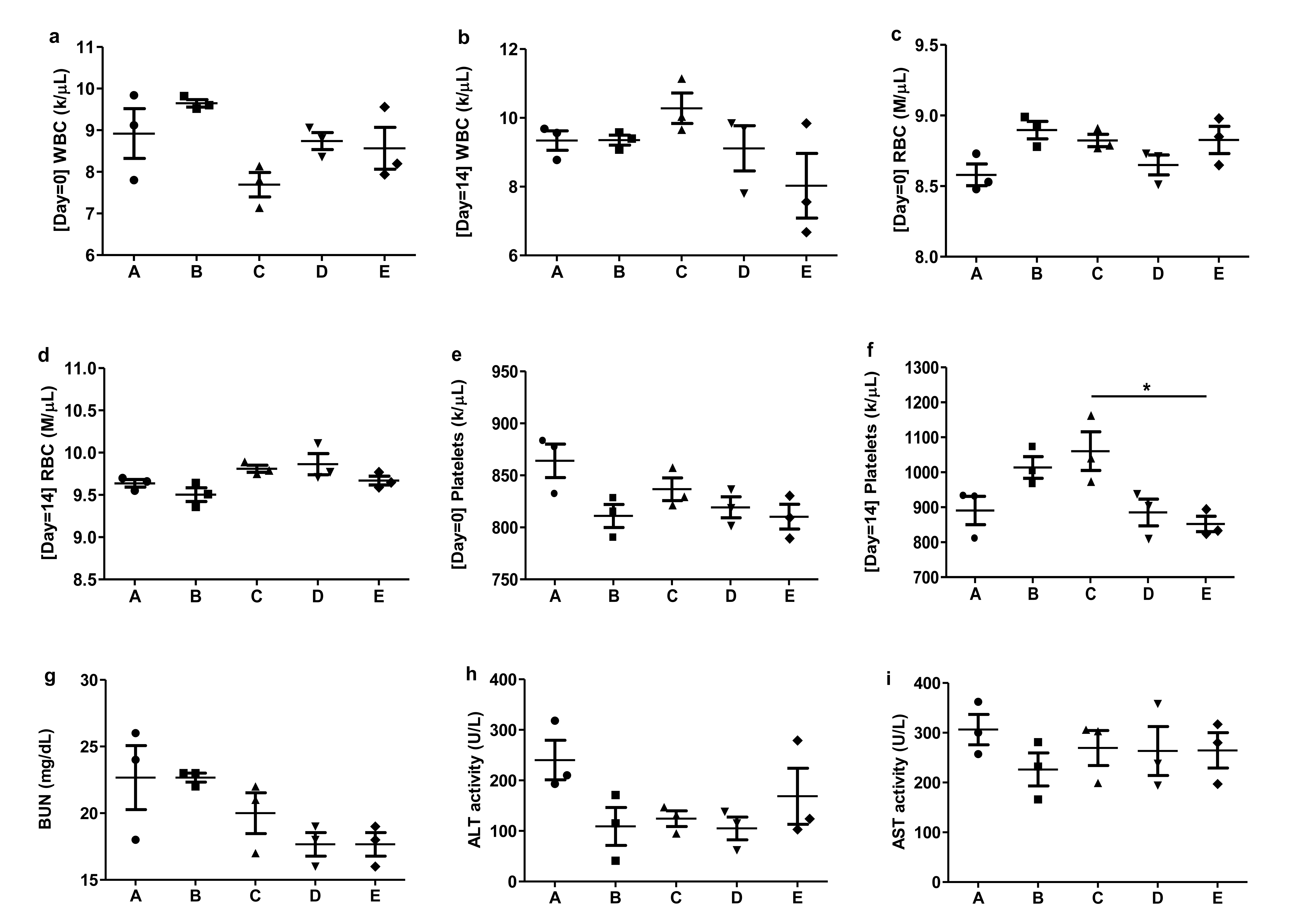

Supplement: Figure S1 — Complete blood count analysis and organ-function tests. PBS (A), Δ12-PGJ3 at (0.025 (B) and 0.050 (C) mg/kg body weight/day) and ZK118182 (0.025 (D) and 0.050 (E) mg/kg body weight/day) were injected intraperitoneally into C57BL/6 mice. Post 0 and 14 days of administration, the blood was collected and analyzed. Panels a, c and e indicate levels of WBC, RBC and platelets at day = 0, respectively. Panels b, d and f indicate levels of WBC, RBC and platelets at day 14, respectively. Panels g-i indicate single slide chemical analyses were carried-out after 14 days of treatments. The blood was collected and plasma analyzed for BUN (g), ALT (h) and AST (i). The data shown here are mean ± SEM (n = 3) and statistical significance represented as *(p≤0.05). (TIF) [file pone.0080622.s001.tif]
